# Altered Immunoregulation in Rheumatoid Arthritis: The Role of Regulatory T Cells and Proinflammatory Th17 Cells and Therapeutic Implications

**DOI:** 10.1155/2015/751793

**Published:** 2015-03-30

**Authors:** Alessia Alunno, Mirko Manetti, Sara Caterbi, Lidia Ibba-Manneschi, Onelia Bistoni, Elena Bartoloni, Valentina Valentini, Riccardo Terenzi, Roberto Gerli

**Affiliations:** ^1^Rheumatology Unit, Department of Medicine, University of Perugia, 06132 Perugia, Italy; ^2^Department of Experimental and Clinical Medicine, Section of Anatomy and Histology, University of Florence, 50134 Florence, Italy

## Abstract

In recent years several studies investigated the role of T lymphocyte subpopulations in the pathogenesis of rheumatoid arthritis (RA). Pathogenic Th17 cells mediate pannus growth, osteoclastogenesis, and synovial neoangiogenesis; hence they are key players in the development of the disease. On the other hand, regulatory T (Treg) cells are a T cell subset whose peculiar function is to suppress autoreactive lymphocytes. The imbalance between Th17 and Treg cells has been identified as a crucial event in the pathogenesis of RA. In addition, the effects of currently employed RA therapeutic strategies on these lymphocyte subpopulations have been extensively investigated. This review article aims to discuss current knowledge on Treg and Th17 cells in RA and possible implications of their therapeutic targeting in this disorder.

## 1. Introduction

Rheumatoid arthritis (RA) is a chronic inflammatory condition characterized by progressive articular cartilage destruction and bone resorption [1]. Although articular involvement dominates the clinical picture in RA, a subgroup of patients may experience extra-articular manifestations such as pulmonary disease that significantly worsen disease prognosis [2, 3].

The breaking of self-tolerance is a hallmark of the disease leading to the production of autoantibodies such as rheumatoid factor and anticyclic citrullinated peptide antibodies. Besides the crucial and well-characterized role of B lymphocytes in RA pathogenesis, also T cells are active players in this scenario. In normal conditions, Th1 and Th2 cells mediate immune responses against intracellular and extracellular pathogens, respectively. However, both cell subsets may participate in the development of autoimmunity, and Th2 cells are also involved in allergy and asthma. In the last decades, the Th1/Th2 immune response paradigm was challenged following the identification of additional T cell subsets with either effector or regulatory activity [4]. In addition, the observation of Th cell flexibility and plasticity further contributed to increase of the interest on this issue [5]. Among recently identified T cell subsets, including Th9, Th22, and follicular Th cells, Th17 and regulatory T (Treg) cells gained growing scientific interest and have been extensively investigated in several autoimmune/inflammatory disorders. Th17 cells are normally responsible for immune responses against extracellular bacteria and fungi but are also leading actors in the autoimmunity scenario, while Treg cells mediate immune tolerance and attempt to maintain lymphocyte homeostasis.

Their opposite behavior as well as their reciprocal plasticity pointed out the importance of Th17/Treg cell imbalance in the pathogenesis of RA. Indeed, a large amount of data has been published to date, with particular interest on the possible therapeutic targeting of these cells and their products in an attempt to overcome the limitation of currently employed biological therapies.

The aim of this paper is the critical discussion of current knowledge on Treg and Th17 cells in RA and possible implications of their therapeutic targeting in this disorder.

## 2. Treg Cells in RA Peripheral Blood and Synovium

Since their first identification in mice and humans [6], Treg cells have been extensively investigated in several autoimmune disorders including RA. Treg cells can be divided in two subgroups: natural Treg cells, generated in the thymus in the early phases of life, and inducible Treg cells that originate in the periphery throughout the entire life. The peculiar function of Treg cells is that of preventing autoimmunity via the suppression of autoreactive lymphocytes. Such effect is mediated either via cell-cell contact or via secretion of soluble molecules including interleukin- (IL-) 10 and transforming growth factor- (TGF-) *β*. As far as Treg phenotype is concerned, although Treg cells were initially identified as CD4^+^CD25^high^ T cells, recent data suggest that the expression of CD25 on the cell surface is not mandatory to confer regulatory properties. In fact, the transcription factor FoxP3 is currently the most specific Treg cell marker and is able to ensure suppressive activity independently on CD25 coexpression [7–9].

In the last decade, a consistent number of studies investigated the number, phenotype, and function of Treg cells in the peripheral blood, synovial fluid, and synovial membrane of RA patients (Table 1). It is important to note that, besides a general agreement on Treg cell enrichment in RA synovial fluid [10–18], conflicting results have been reported concerning Treg cell proportion in RA peripheral blood. In particular, most studies observed reduced circulating Treg cell percentages in RA compared to healthy individuals [11, 16, 19–23], while some other studies reported either an increase [12, 24] or similar cell percentages compared to normal controls [10, 13, 14, 18, 25–27] or patients with osteoarthritis (OA) [17]. These apparently paradoxical discrepancies deserve some consideration. In earlier studies, Treg cells were defined as CD4^+^CD25^high^ cells and FoxP3 coexpression was not routinely assessed [10–14, 27, 28]. However, in 2008 Han and coworkers pointed out that CD25^high^ cells include a high proportion of FoxP3^−^ cells that cannot be classified as Treg cells [24]. In fact, CD25 can be expressed also by recently activated cells that do not coexpress FoxP3 [29]. Hence, the higher cell percentages of Treg cells reported by some studies may reflect a contamination of activated cells with consequent reduced number of the overall FoxP3 expression among RA peripheral blood CD25^high^ cells compared to healthy individuals [24]. In addition, other surface markers that allow the distinguishing of different subsets of natural and induced Treg cells, such as Neuropilin-1 [30] or Helios [31], have not been investigated in RA.

Concerning synovial fluid, FoxP3 mRNA expression in CD25^high^ T cells is higher in both RA and OA compared to CD25^−^ effector cells [13, 15, 17], as well as in total RA synovial fluid mononuclear cells compared to total peripheral blood mononuclear cells [16]. However, flow cytometry data on FoxP3^+^ cell percentage among CD25^high^ synovial fluid cells are not available.

Taken together, these observations allowed the conclusion that although some RA patients display an expansion of CD25^high^ cells in peripheral blood or synovial fluid, the identification of real Treg cells, namely, those FoxP3^+^, should be recommended to provide more precise cell percentages and allow a comparison between different studies.

Finally, studies performing synovial immunohistochemical staining to detect FoxP3 consistently reported that Treg cells are diffusely present in the hyperplastic synovial lining and in the sublining tissue and that their number increases in parallel with the worsening of inflammation [32–34]. Furthermore, the only study that quantified CD25^high^ Treg cells by flow cytometry in cell suspensions obtained from RA synovial biopsies showed that Treg cell percentage is significantly higher in this compartment compared to peripheral blood and significantly lower compared to synovial fluid [17].

The evidence of increased percentages of Treg cells both in RA synovial fluid and membrane, proven by FoxP3 expression, may suggest a certain attempt to counteract effector T cell response in the target organs of the disease. However, although some* in vitro* studies reported that suppressive activity appears to be, at least partially, preserved in Treg cells from peripheral blood [12, 14, 22, 24, 28] and synovial fluid [10–15, 28, 33], it should be borne in mind that this may be an artifact due to the removal of Treg cells from a proinflammatory microenvironment. Therefore, any speculation about the function of Treg cells* in vivo* in RA should be performed with caution.

Studies attempting to identify correlations between Treg cells and clinical/serological features of the disease yielded often contradictory results [11, 12, 19–21, 24, 26, 32]. An inverse relationship between disease activity score on 28 joints (DAS28) and the percentage of circulating CD25^high^ Treg cells has been reported [19–21]. On the other hand, however, a surprisingly higher percentage of FoxP3^+^ cells were also observed among CD25^high^ Treg cells from active RA patients [19, 26].

Concerning synovial tissue Treg cells, Behrens et al. described a direct relationship between synovial T-bet/FoxP3 mRNA ratio and DAS28, suggesting a quantitative Treg deficiency in RA target tissue [32].

As far as acute phase reactants are concerned, such as erythrocyte sedimentation rate (ESR) and C-reactive protein (CRP), either an inverse relationship or no association with Treg cell proportion has been reported [11, 12, 20, 24]. Finally, no association between Treg cell percentage and age, sex, disease duration, rheumatoid factor positivity, and bone erosions has been identified [11, 12, 20, 24].

In conclusion, although often contradictory, the available majority of data points out a reduction of circulating, but an increase of synovial, Treg cells, the latter resulting in a compensatory mechanism to counteract local inflammation.

## 3. Proinflammatory Th17 Cells in RA Peripheral Blood and Synovium

Th17 cells represent a distinct effector T cell subset characterized by the expression of the retinoic acid-related orphan receptor- (ROR-) *γ*t and the production of IL-17 family members, IL-21, and IL-22 [62]. The IL-17 family consists of six members, from IL-17A to IL-17F. To date, IL-17 refers to IL-17A, which is the founding member of the IL-17 family.

The polarization of a naïve T cell towards a Th17 cell is a multistep process requiring a peculiar cytokine milieu that includes IL-6, TGF-*β*, IL-21, IL-1*β*, and IL-23 [5, 63]. Of interest, however, a recent paper provided evidence that pathogenic Th17 cells could be generated also in the absence of TGF-*β* signaling [64].

IL-17 is involved in several physiological and pathological processes as the binding to its receptor leads to the release of proinflammatory mediators, including cytokines, chemokines, and matrix metalloproteinases (MMPs), by the target cell. Therefore, the pathogenic role of IL-17 and IL-17- producing cells has been extensively investigated in a variety of inflammatory and autoimmune diseases [65].

Regarding RA pathogenesis, data from experimental models support the role of IL-17 in pannus growth, RANKL-independent osteoclastogenesis [66–68], and synovial neoangiogenesis [55, 69]. In humans,* in vitro* studies revealed that recombinant IL-17 is able to potentiate the expression of proinflammatory cytokines and prostaglandin E2 in synovial tissue cells, confirming its role in inducing pannus growth and osteoclastogenesis* in vivo* [38, 44, 67]. Furthermore, its proangiogenic potential is also confirmed by the evidence that recombinant IL-17 enhances the production of vascular endothelial growth factor-A in RA synovial fibroblasts [47]. Similarly, when cocultures were arranged with peripheral blood mononuclear cells and synovial Th17 cells instead of recombinant IL-17, a strong enhancement of IL-6, IL-8, MMP-1, and MMP-3 production by RA synovial fibroblasts was observed [54].

At present, several studies evaluating IL-17 and IL-17-producing cells in human RA are available (Table 2) [70].

Concerning IL-17 in biologic fluids, it has been largely investigated since early 2000s. Most studies observed higher concentration of this cytokine in the serum [40, 42, 44, 49, 50, 71] and in the synovial fluid [38–40, 44, 51] of RA patients compared to normal subjects or OA patients. In striking contrast, two studies observed comparable serum levels of this cytokine in RA and controls [42, 59] and another reported reduced concentration of serum IL-17 in RA compared to controls [52].

Such discrepancies in the serum levels of IL-17 may be clarified, at least in part, in those studies in which also circulating Th17 cells were enumerated. When available, indeed, the concentration of serum IL-17 appeared to parallel the number of circulating Th17 cells. In particular, in three studies in which IL-17 was detected at higher concentrations in long-standing RA compared to healthy subjects, the percentage of circulating Th17 cells was also significantly higher [42, 49, 71]. Arroyo-Villa et al. reported reduced levels of both IL-17 and Th17 cells in early RA patients [52], while Fazaa et al. failed to observe any differences in the Th17 cell percentage and IL-17 concentration between patients and controls [59]. Although Shen et al. did not observe any differences in serum IL-17 concentrations, they found higher Th17 cell percentages in RA patients [42].

Additional studies investigated circulating or synovial fluid Th17 cells without the concurrent evaluation of IL-17. In the majority of these, higher percentages of circulating Th17 cells were detected in RA compared to healthy or OA controls [21–23, 36, 47, 53, 54, 57, 60, 61], while in few others Th17 cell proportion in RA was comparable to that of healthy subjects [26, 41, 46]. In synovial fluid, the proportion of Th17 cells was either higher [43, 47], comparable [54], or reduced [41] compared to that found in peripheral blood.

A further complication to this issue comes from the fact that some studies were performed in patients with established RA and others in early RA. In established RA, there is general agreement that circulating Th17 cells are increased in the peripheral blood compared to healthy subjects, even if some authors reported Th17 cell proportions overlapping that of healthy donors [26, 40, 59]. Conversely, in the available studies in early RA, either higher [36, 54] or lower percentages [52] of circulating Th17 cells with respect to healthy subjects were described.

Studies evaluating IL-17 in synovial tissue reported increased immunostaining as well as mRNA expression in RA synovial membrane compared to OA [37, 38, 43, 44, 47, 48, 51, 56, 58]. Although in RA synovium IL-17^+^ cells are mostly CD4^+^ cells [38] mainly localized in the T cell area [37]; also macrophages and mast cells appear to be a local source of IL-17 [48, 51, 58]. Moreover, a recent study identified IL-17^+^FoxP3^+^ T cells in human RA synovial tissue [72]. This observation is in line with data obtained in experimental arthritis reporting that Th17 cells can arise from Treg cells following FoxP3 loss. These so-called exFoxP3 Th17 cells appear to be more pathogenic than those originating from naïve T cells [72].

Finally, several studies also investigated possible correlations between IL-17 or Th17 cell proportion and disease activity. Most studies agree that serum IL-17 concentration [50, 71, 73] and circulating Th17 cell percentage [21, 53, 61] positively correlate with DAS28. In addition, synovial IL-17 staining was found to be directly correlated with DAS28 [47, 56]. To note, a direct correlation between synovial fluid Th17 cell percentage and ultrasound power Doppler signal in the corresponding joint has been also reported [47]. Finally, synovial fluid IL-17 was correlated with the degree of intimal lining layer hyperplasia in paired synovial samples [73]. Concerning serological features of the disease, serum IL-17 levels appear to be directly correlated with both CRP and ESR [45, 71], and synovial fluid Th17 cell proportion appears to be directly correlated with CRP [47].

In conclusion, Th17 cells and their products appear to be leading players in RA pathogenesis, and an overall increase of both has been widely demonstrated. These findings, together with the aforementioned impairment of Treg cells, depict an intriguing pathogenic scenario worth targeting for therapeutic purposes.

## 4. The Effects of Different RA Therapeutic Approaches on Treg and Th17 Cells

**Figure 1:**
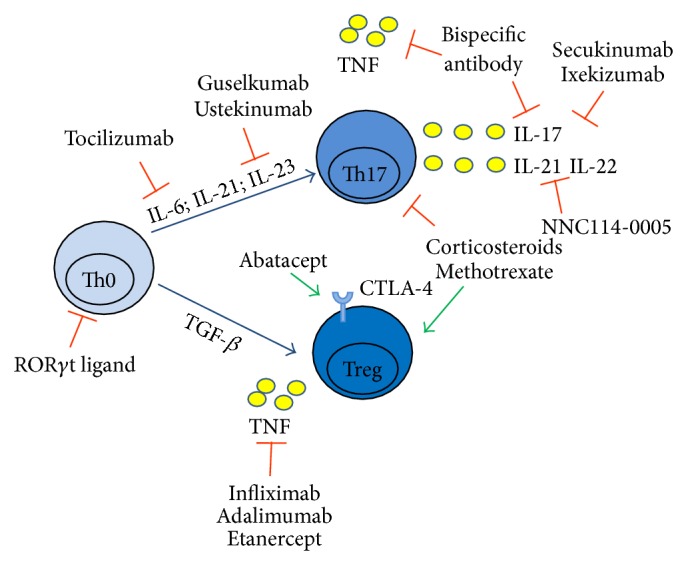
Therapeutic targeting of Treg and Th17 cells in rheumatoid arthritis (RA). The figure displays currently employed therapeutic approaches in RA for which an effect on Treg and Th17 cells has been reported in the literature (see text for details). Other compounds depicted in the figure are currently under investigation in RA or have been tested in experimental models of the disease. CTLA-4: cytotoxic T lymphocyte antigen 4; IL: interleukin; TGF-*β*: transforming growth factor-*β*; TNF: tumor necrosis factor.

The growing number of studies supporting Treg/Th17 cell imbalance as pathogenic mechanism in RA prompted to investigate the effect of currently employed therapies on these cell subsets (Figure 1).

### 4.1. Corticosteroids and Disease Modifying Antirheumatic Drugs

Corticosteroids (CS) are well known modulators of Treg cells as widely documented in asthma [74]; however very few data on this issue are available in RA. Recently, de Paz et al. published two interesting studies that linked higher percentages of circulating CD4^+^CD25^high^ Treg cells and CD25^−^FoxP3^+^ T cells to CS treatment in RA [75, 76]. The latter were already identified by Raghavan et al. in RA synovial fluid [33]. CD25^−^FoxP3^+^ T cell expansion has been also found in systemic lupus erythematosus (SLE) [77], but its suppressive activity is a matter of debate [78]. We recently demonstrated that, among CD25^−^ T cells, those coexpressing glucocorticoid-induced tumor necrosis factor receptor-related protein (GITR) display consistent suppressive activity and are expanded in SLE and primary Sjögren syndrome (pSS) [79–81]. Intriguingly, since GITR is a CS-inducible molecule, it would be of great interest to clarify whether the increase of CD25^−^FoxP3^+^ cells that de Paz et al. observed in CS-treated RA patients [75, 76] was due to a selective increase of CD25^−^FoxP3^+^GITR^+^ rather than CD25^−^FoxP3^+^GITR^−^ T lymphocytes.

In striking contrast, a reduction of FoxP3 staining in synovial samples obtained from RA patients before and after intra-articular CS treatment, in parallel with the general reduction in inflammation, has been also described [33]. In light of the observation by Komatsu et al. concerning the presence of FoxP3^+^IL-17^+^ cells in RA synovium [72], an intriguing explanation for the synovial FoxP3^+^ cell reduction induced by CS may be a selective depletion of exFoxP3^+^ Th17 cells.

Th17 cells appear to be major players in the context of CS resistance in inflammatory diseases. In particular, a recent study revealed that pathogenic proinflammatory Th17 cells could be identified by their distinct phenotype (CCR6^+^CXCR3^hi^CCR4^lo^CCR10^−^CD161^+^) that includes the stable expression of P-glycoprotein/multi-drug resistance type 1. To note, when these cells were isolated from healthy subjects and cultured with CS, they resulted in being refractory to these compounds [82]. In this setting, we reported that IL-17-producing CD3^+^CD4^−^CD8^−^ double negative T cells isolated from pSS patients, but not those from healthy subjects, are insensitive to dexamethasone* in vitro* [83]. On this basis, the selective depletion of CS-resistant Th17 cells, on one hand, and the understanding of molecular mechanisms responsible for CS resistance of double negative T cells, in an attempt to revert it, on the other hand, are intriguing issues.

In the matter of disease modifying antirheumatic drugs (DMARDs), there are no studies reporting* in vivo* Treg or Th17 cell modulation exerted by these compounds in RA patients.

A recent study evaluated ovalbumin-immunized mice treated with methotrexate (MTX), cyclophosphamide (CTX), or a combination of the two drugs. It was observed that MTX+CTX, but not each compound in monotherapy, induced Treg skewing and Th17 suppression by interference with dendritic cell maturation and antigen presenting ability [84].

To date, a few studies reported MTX* in vitro* effects in RA [36, 85–87]. The exposure of peripheral blood mononuclear cells isolated from RA patients to this compound led to a consistent upregulation of FoxP3, TGF-*β*, and IL-10 in CD4^+^ cells, an enhancement of Treg cell suppressive activity [85], and a reduction of IL-17 mRNA [86]. In addition, our group has demonstrated that MTX is able to downregulate IL-17 and related cytokines, namely, IL-6, IL-22, and IL-23, but not IL-21, in culture supernatants of RA peripheral blood mononuclear cells [85]. Finally, a reduction of Th17 cell percentage following MTX* in vitro* exposure has been also described in patients with early, but not long-standing, RA [36]. Of interest, the MTX-induced upregulation of FoxP3 in peripheral blood mononuclear cells isolated from RA patients was not seen in mononuclear cells from healthy subjects [85, 87].

About hydroxychloroquine, the only study available to date reported that the addition of this compound to RA peripheral blood mononuclear cells* in vitro* is able to reduce IL-17, IL-6, and IL-22 secretion in culture supernatants [88].

### 4.2. Antitumor Necrosis Factor Agents

In the last decade, a growing number of studies underscored the effects of biologic agents on Treg and Th17 cells in RA. Concerning Treg cells, the possible role of tumor necrosis factor (TNF) blockers on this cell subset was initially reported by Ehrenstein et al. in 2004 [35]. In fact, they observed that treatment with infliximab, a chimeric monoclonal antibody against TNF, was able to increase the percentage of circulating CD4^+^FoxP3^+^ cells and to revert the defective suppressive activity of CD25^high^ Treg cells [35]. To note, however, the increase of circulating CD4^+^FoxP3^+^ cells induced by infliximab was due to a selective upregulation of the FoxP3 transcription factor in CD25^−^ rather than CD25^high^ T cells [89]. Hence, in a coculture system of RA CD25^−^ and CD25^high^ T cells, the apparent restoration of CD25^high^ Treg cell suppressive activity following infliximab treatment was an artifact due to increased percentage of suppressive FoxP3^+^ cells among the CD25^−^ fraction.

Subsequently, several studies attempted to investigate the effect of other commercially available TNF blockers on RA Treg cells. Concerning the human monoclonal antibody adalimumab, while two studies failed to observe any differences in Treg cell percentage before and after treatment [27, 90], three other groups reported increased percentages of circulating CD25^high^ FoxP3^+^ Treg cells either in accordance with [91] or independently from clinical response to adalimumab [92, 93]. Moreover, Treg cells isolated from RA patients with good clinical response to adalimumab appear to exert a more pronounced suppressive activity [91, 93]. Increased FoxP3 expression among CD4^+^ lymphocytes has been described in patients treated with etanercept, a fusion protein acting as TNF inhibitor [94], but these data were not confirmed in other studies evaluating the* in vivo* effects of this compound on RA Treg cells [90, 91].

The exact molecular mechanism underlying the possible inhibitory effect exerted by TNF on Treg cells, thus explaining their modulation by TNF blockers, was only recently clarified. Valencia et al., indeed, observed that TNF is directly responsible for the impaired suppressive activity of RA CD25^high^ Treg cells, as it determines a consistent reduction of FoxP3 mRNA, required to convey a regulatory activity [95]. This effect appeared to be mediated through TNFRII that is constitutively expressed by Treg cells [95]. More recently, Nie et al. demonstrated that FoxP3 transcriptional activity and Treg cell suppressive function are regulated by TNF-dependent dephosphorylation of the FoxP3 DNA-binding domain (Ser418 in the C-terminal DNA-binding domain) [96, 97]. This abnormal dephosphorylation of FoxP3 in RA Treg cells is due to the ubiquitous enzyme protein phosphatase 1 that is induced by TNF through the IKK–NF-*κ*B pathway. Of interest, treatment of RA patients with TNF blockers decreased protein phosphatase 1 expression, increased FoxP3 phosphorylation, and, in consequence, restored Treg cell suppressive activity.

As far as the IL-17 axis is concerned, there is general agreement that infliximab or adalimumab-treated RA patients display lower percentages of circulating Th17 cells [45, 91, 92, 98]. In striking contrast, increase of circulating Th17 cells in adalimumab-treated* versus* anti-TNF-naïve RA patients, independently of clinical response, has been observed [46]. However, Th17 cells of adalimumab-treated patients with inactive disease displayed very low levels of the chemokine receptor CCR6, allowing postulating that although increased, Th17 cells are not able to migrate to RA target tissue in these patients [46]. Etanercept, instead, appears to affect neither the percentage of circulating Th17 cells [91] nor the concentration of serum IL-17 but is able to decrease serum IL-23 concentration [40].

In light of these findings, Treg cell specific targeting may be an additional rationale to employ TNF blockers in RA. As far as Th17 cells, although conclusive data are still lacking, the possible biases due to concurrent treatments that affect T cells, mainly CS, should be taken into account, and it is conceivable that prospective studies may help to shed additional light on this issue.

### 4.3. Abatacept

The first description of cytotoxic T lymphocyte antigen 4 (CTLA-4) abnormalities in functionally defective Treg cells in RA dates back to 2008, when reduced levels and increased internalization rate of CTLA-4 were described in Treg cells from RA patients compared to those from healthy subjects [99]. Since the artificial induction of CTLA-4 expression on RA Treg cells restored their suppressive capacity and CTLA-4 blockade on healthy Treg cells hampered their function, the authors speculated that CTLA-4 on RA Treg cells may represent a potential therapeutic target to directly interfere with these cells.

The mechanism underlying the impairment of CTLA-4 system in RA was unmasked in a recent study showing that downregulation of CTLA-4 expression in RA Treg cells is caused by methylation of a newly identified NF-AT binding site within the CTLA-4 gene promoter [100]. Of particular interest, this finding may also help to understand, at least in part, why RA Treg cells are functionally defective. In fact, the binding of CD80/CD86 on dendritic cells by CTLA-4 expressed on normal Treg cells induces the activation of the indoleamine 2,3-dioxygenase (IDO) enzyme [101, 102]. In RA, the reduced CTLA-4 gene transcriptional activity, due to the aforementioned epigenetic modification, prevents the activation of the IDO immune-modulatory pathway in antigen presenting cells (APCs) and, therefore, contributes to defective Treg cell function [101, 102].

On this basis, the effects on RA Treg cells of abatacept, a CTLA-4 immunoglobulin currently employed to treat this disorder, have been subsequently investigated. CTLA-4 immunoglobulin exerts its immune-modulatory effect via agonism of CD80/CD86 expressed by APCs by blocking the second signal required for the activation of effector T lymphocytes as well as licensing APCs to express IDO [103, 104].

Either a reduction [105, 106], no modification [107], or an increase [108] of circulating CD25^high^ and FoxP3^+^ Treg cells in abatacept-treated RA patients has been reported. Furthermore, abatacept treatment in RA appears to restore, at least in part, the defective suppressive activity of circulating Treg cells [105, 107], but this observation was not confirmed by* in vitro* experiments with synovial fluid Treg cells [106].

To date, only two studies assessed the possible effect of abatacept on circulating Th17 cells, but they obtained opposite results. In fact, Scarsi et al. reported a reduction of the proportion of Th17 cells [108], while Pieper et al. did not observe any modification in this cell subset following abatacept therapy [106].

The involvement of CTLA-4 in Treg cell biology is an intriguing issue. However, the targeting of this molecule did not provide the expected results. In addition, it is unclear how CTLA-4 may participate in Th17 cell balance, and the few data available do not allow the drawing of definitive conclusions.

### 4.4. Tocilizumab

The notion that IL-6 is the key cytokine that determines the commitment of a naïve T lymphocyte towards a Treg or a Th17 cell prompted the investigation of the effects of its blockade on Treg/Th17 cell balance in autoimmune diseases, including RA. Evidence from experimental models of RA pointed out that early treatment with anti-IL-6 receptor antibody led to a reduced frequency of circulating Th17 cells and, therefore, to a milder clinical picture [109]. Of interest, if treatment was administered later in the course of the disease, these effects were no longer detectable [109]. In line with these findings, a study investigating the effects of the commercially available anti-IL-6 receptor antibody tocilizumab in early RA revealed a similar reduction of circulating Th17 cells after three months of treatment [110]. Such decrease, however, was confirmed only in another study enumerating Th17 cell frequencies after four months of tocilizumab treatment [22]. In fact, other studies did not observe any differences in Th17 cell percentages up to 6 months after treatment [111, 112].

Concerning Treg cells, progressive increase of their proportion starting from the first month of therapy with subsequent stability overtime in the 12-month follow-up has been described in all the available studies [22, 112, 113] except one that reported a surprising reduction of circulating Treg cells induced by tocilizumab in early RA [110].

On this basis, it appears that IL-6 blockade rebalances Treg/Th17 cell ratio in RA affecting at least one of these T cell subsets, Treg cells. An intriguing perspective may be to concurrently target IL-6 and other cytokines involved in Th17 cell polarization to clarify whether this approach may provide additional clinical benefit.

### 4.5. IL-17 Targeted Therapies and Other Future Perspectives

Taking the well-characterized pathogenic role of IL-17 axis in autoimmune diseases, in recent years a variety of compounds targeting this system at different levels are being intensively investigated [114]. Although the direct blockade of IL-17 with either fully human or humanized monoclonal antibodies, such as secukinumab and ixekizumab, respectively, may be the most straightforward approach for RA, results from clinical trials revealed lower clinical efficacy than expected for these compounds. This may be explained, at least in part, by the heterogeneous expression of IL-17 in RA synovial tissue and may be overcome by patient stratification based on IL-17 expression [58].

Alternatively, the targeting of molecules involved upstream in the process of Th17 cell generation may be considered. In this setting, ustekinumab, an anti-p40 subunit of IL-12/IL-23 monoclonal antibody currently employed for the treatment of plaque psoriasis, is under investigation in chronic inflammatory arthritides [115, 116]. Guselkumab, a human IL-23-specific monoclonal antibody recently evaluated in psoriasis [117], may also find a therapeutic application in RA as well as NNC114-0005, an anti-IL-21 antibody that was investigated in RA in phase I trials [118].

In addition, a very intriguing therapeutic approach is represented by the interference with Th17 cell generation using small molecules able to modulate ROR*γ*t expression. An elegant study recently showed an improvement of neurological symptoms in an experimental model of multiple sclerosis treated with the high-affinity synthetic ligand SR1001 specific to both ROR*α* and ROR*γ*t that inhibits Th17 cell differentiation and function [119].

In line with the current knowledge on the role of Treg and Th17 cells in RA pathogenesis, however, the identification of a therapeutic approach able to rebalance their ratio concurrently targeting both cell subsets may be the most suitable choice. In this setting, the blockade of different cytokine systems by bispecific antibodies is an intriguing possibility.

In a mouse model of RA, combined TNF/IL-17 inhibition resulted in virtual abrogation of synovitis similarly to anti-TNF monotherapy, but with superior effect on bone erosion compared to anti-TNF or anti-IL-17 monotherapies [120]. On the basis of this observation, the same group recently developed and characterized a bispecific antibody to target both TNF and IL-17 and tested this compound in RA fibroblast-like synoviocytes (FLS)* in vitro* [120]. When RA-FLS were stimulated with either TNF or IL-17 alone and treated with the corresponding blocking antibody or the bispecific one, a similar reduction of proinflammatory cytokine release was observed in the three conditions. Of great interest, however, when RA-FLS were stimulated with both TNF and IL-17, the bispecific antibody exhibited a greater inhibitory effect on proinflammatory cytokine release compared to single blocking antibodies [120]. Since RA-FLS are exposed to a heterogeneous proinflammatory milieu* in vivo*, this therapeutic approach seems to represent an intriguing option worth investigating further.

Finally, possible effects of B-cell targeted therapies on Treg and Th17 cells deserve some considerations. The initial observation by Mélet et al. that the anti-CD20 antibody rituximab induces a consistent depletion of circulating T cells, mainly those CD4^+^, in RA patients prompted the investigation of the specific T cell subset possibly affected by this compound as well as the mechanism at the basis of this effect [121]. Although Treg cells do not appear to be affected by rituximab [122, 123], a recent investigation reported that rituximab was able to reduce, at least in rheumatoid synovium, the Th17, but not Th1, response [122]. Of interest, a subset of IL-17-producing cells isolated from the peripheral blood of healthy subjects coexpresses CD20, and these CD20^+^IL-17^+^ T lymphocytes are expanded in the circulation of RA patients [124].

These intriguing observations further underscore the therapeutic rationale for rituximab in RA, as it appears able to target not only the pathogenic B-cell compartment, but also the T cell compartment in this disease.

In conclusion, most of the currently employed therapeutic approaches in RA appear able also to target Treg/Th17 cells and this contributes to their clinical efficacy. It would be of interest, however, to verify whether the selective targeting of these cell subsets may provide additional clinical benefit, thus further supporting the rationale to include these compounds in clinical practice.

## 5. Conclusions

In conclusion, a large body of evidence supports the concept that an imbalance between Treg and Th17 cells is a crucial aspect in the pathogenesis of RA. Although often contradictory, most studies agree that an overall depletion of Treg and a parallel increase of Th17 cells in the peripheral blood and target organs could be detected in RA patients. In addition, intrinsic cell abnormalities, involving genetic and epigenetic modifications, may explain the defective suppressive activity of RA Treg cells.

Currently employed therapeutic strategies, mostly biotechnologic agents, appear to actively interfere with Treg and Th17 cells and restore either their correct proportion or, for Treg cells, their suppressive function. However, although intriguing, this evidence needs to be confirmed in larger prospective studies.

The new therapeutic compounds currently under investigation in RA, such as anti-IL-17 antibodies or anti-TNF/IL-17 bispecific antibodies, represent a promising option and studies aimed at characterizing their activity on Treg and Th17 cells will be of great interest.

Finally, the increasing knowledge on Treg cell markers and selective isolation procedures may allow directly employing* ex vivo* expanded Treg cells for therapeutic purposes in RA, eventually aiming at the reset of the immune system and restoration of tolerance [125, 126].

## Figures and Tables

**Table 1:** Studies assessing regulatory T (Treg) cell number and function in the peripheral blood, synovial fluid, and synovial tissue of patients with rheumatoid arthritis.

Authors (year)	Reference	Treg cells			FoxP3 assessment	Treg suppressive activity	
		PB	SF	SM		PB	SF
Cao et al. (2003)	[10]	= to HD	↑versus RA PB	N.A.	No	N.A.	Present
Cao et al. (2004)	[11]	↓ versus HD	↑versus RA PB	N.A.	No	N.A.	Present
Ehrenstein et al. (2004)	[35]	N.A.	N.A.	N.A.	Yes	Impaired	N.A.
van Amelsfort et al. (2004)	[12]	↑ versus HD	↑ versus RA PB	N.A.	No	Present	↑ versus RA PB
Möttönen et al. (2005)	[13]	= to HD	↑ versus RA PB	N.A.	Yes	N.A.	Present
Liu et al. (2005)	[14]	= to HD	↑ versus RA PB	N.A.	No	Present	Present
Cao et al. (2006)	[15]	N.A.	↑ versus RA PB	N.A.	Yes	N.A.	Present
Dombrecht et al. (2006)	[27]	= to HD	N.A.	N.A.	No	N.A.	N.A.
van Amelsfort et al. (2007)	[28]	N.A.	N.A.	N.A.	No	Present	Present
Behrens et al. (2007)	[32]	N.A.	N.A.	Present	Yes	N.A.	N.A.
Lin et al. (2007)	[25]	= to HD	N.A.	N.A.	Yes	N.A.	N.A.
Jiao et al. (2007)	[16]	↓ versus HD	↑ versus RA PB	N.A.	Yes	N.A.	N.A.
Han et al. (2008)	[24]	↑ versus HD	N.A.	N.A.	Yes	Present	N.A.
Raghavan et al. (2009)	[33]	N.A.	N.A.	Present	Yes	N.A.	Present
Sempere-Ortells et al. (2009)	[19]	↓ versus HD	N.A.	N.A.	Yes	N.A.	N.A.
Dejaco et al. (2010)	[18]	= to HD	↑ versus RA PB	N.A.	No	N.A.	N.A.
Kawashiri et al. (2011)	[20]	↓ versus HD	N.A.	N.A.	No	N.A.	N.A.
Lina et al. (2011)	[23]	↓ versus HD	N.A.	N.A.	Yes	N.A.	N.A.
Niu et al. (2012)	[21]	↓ versus HD	N.A.	N.A.	Yes	N.A.	N.A.
Xq et al. (2012)	[34]	N.A.	N.A.	Present	↑ versus OA-SM	N.A.	N.A.
Samson et al. (2012)	[22]	↓ versus HD	N.A.	N.A.	Yes	Present	N.A.
Ji et al. (2013)	[26]	= to HD	N.A.	N.A.	Yes	N.A.	N.A.
Moradi et al. (2014)	[17]	= to OA	↑ versus RA PB	Present	Yes	N.A.	N.A.
Guggino et al. (2015)	[36]	↓ versus HD	N.A.	N.A.	Yes	N.A.	N.A.

PB: peripheral blood; SF: synovial fluid; SM: synovial membrane; RA: rheumatoid arthritis; OA: osteoarthritis; HD: healthy donors; N.A.: not applicable.

**Table 2:** Studies assessing Th17 cells and IL-17 in the peripheral blood, synovial fluid, and synovial tissue of patients with rheumatoid arthritis.

Authors (year)	Reference	Th17 cells			IL-17 concentration	
		PB	SF	SM	Serum	SF
Chabaud et al. (1999)	[37]	N.A.	N.A.	↑ versus OA	N.A.	N.A.
Kotake et al. (1999)	[38]	N.A.	N.A.	↑ versus OA	N.A.	↑ versus OA
Ziolkowska et al. (2000)	[39]	N.A.	N.A.	N.A.	N.A.	↑ versus OA
Kageyama et al. (2007)	[40]	N.A.	N.A.	N.A.	↑ versus HD	↑ versus OA
Yamada et al. (2008)	[41]	= versus HD	↓ versus RA PB	N.A.	N.A.	N.A.
Shen et al. (2009)	[42]	↑ versus HD	N.A.	N.A.	= versus HD	N.A.
Shahrara et al. (2008)	[43]	N.A.	↑ versus RA PB	↑ versus OA	N.A.	N.A.
Moran et al. (2009)	[44]	N.A.	N.A.	↑ versus OA	↑ versus OA	↑ versus OA
Shen et al. (2010)	[45]	↑ versus HD	N.A.	N.A.	↑ versus HD	N.A.
Aerts et al. (2010)	[46]	= versus HD	N.A.	N.A.	N.A.	N.A.
Gullick et al. (2010)	[47]	↑ versus HD	↑ versus RA PB	Present	N.A.	N.A.
Hueber et al. (2010)	[48]	N.A.	N.A.	↑ versus OA	N.A.	N.A.
Chen et al. (2011)	[49]	↑ versus HD	N.A.	N.A.	↑ versus HD	N.A.
Lina et al. (2011)	[23]	↑ versus HD	N.A.	N.A.	N.A.	N.A.
Metawi et al. (2011)	[50]	N.A.	N.A.	N.A.	↑ versus HD	↑ versus OA
Suurmond et al. (2011)	[51]	N.A.	N.A.	= versus OA	N.A.	↑ versus OA
Samson et al. (2012)	[22]	↑ versus HD	N.A.	N.A.	N.A.	N.A.
Arroyo-Villa et al. (2012)	[52]	↓ versus HD	N.A.	N.A.	↓ versus HD	N.A.
Zhang et al. (2012)	[53]	↑ versus HD	N.A.	N.A.	N.A.	N.A.
van Hamburg et al. (2013)	[54]	↑ versus HD	= versus RA PB	N.A.	N.A.	N.A.
Niu et al. (2012)	[21]	↑ versus HD	N.A.	N.A.	N.A.	N.A.
Kim et al. (2013)	[55]	↑ versus HD and OA	N.A.	N.A.	↑ versus HD	N.A.
Li et al. (2013)	[56]	N.A.	N.A.	Present	N.A.	N.A.
Henriques et al. (2013)	[57]	↑ versus HD	N.A.	N.A.	N.A.	N.A.
van Baarsen et al. (2014)	[58]	N.A.	N.A.	Present	N.A.	N.A.
Fazaa et al. (2014)	[59]	= versus HD	N.A.	N.A.	= versus HD	N.A.
Sarkar and Fox (2010)	[60]	↑ versus OA	Absent	N.A.	N.A.	N.A.
Miao et al. (2014)	[61]	↑ versus HD	N.A.	N.A.	N.A.	N.A.
Guggino et al. (2015)	[36]	↑ versus HD	N.A.	N.A.	N.A.	N.A.

PB: peripheral blood; SF: synovial fluid; SM: synovial membrane; RA: rheumatoid arthritis; OA: osteoarthritis; HD: healthy donors; N.A.: not applicable.
